# First Approximations of Prescribed Fire Risks Relative to Other Management Techniques Used on Private Lands

**DOI:** 10.1371/journal.pone.0140410

**Published:** 2015-10-14

**Authors:** Dirac Twidwell, Carissa L. Wonkka, Michael T. Sindelar, John R. Weir

**Affiliations:** 1 Department of Agronomy and Horticulture, University of Nebraska-Lincoln, Lincoln, Nebraska, United States of America; 2 Department of Natural Resource Ecology and Management, Oklahoma State University, Stillwater, Oklahoma, United States of America; West Chester University of Pennsylvania, UNITED STATES

## Abstract

Fire is widely recognized as a critical ecological and evolutionary driver that needs to be at the forefront of land management actions if conservation targets are to be met. However, the prevailing view is that prescribed fire is riskier than other land management techniques. Perceived risks associated with the application of fire limits its use and reduces agency support for prescribed burning in the private sector. As a result, considerably less cost-share support is given for prescribed fire compared to mechanical techniques. This study tests the general perception that fire is a riskier technique relative to other land management options. Due to the lack of data available to directly test this notion, we use a combination of approaches including 1) a comparison of fatalities resulting from different occupations that are proxies for techniques employed in land management, 2) a comparison of fatalities resulting from wildland fire versus prescribed fire, and 3) an exploration of causal factors responsible for wildland fire-related fatalities. This approach establishes a first approximation of the relative risk of fatality to private citizens using prescribed fire compared to other management techniques that are readily used in ecosystem management. Our data do not support using risks of landowner fatalities as justification for the use of alternative land management techniques, such as mechanical (machine-related) equipment, over prescribed fire. Vehicles and heavy machinery are consistently leading reasons for fatalities within occupations selected as proxies for management techniques employed by ranchers and agricultural producers, and also constitute a large proportion of fatalities among firefighters. Our study provides the foundation for agencies to establish data-driven decisions regarding the degree of support they provide for prescribed burning on private lands.

## Introduction

Prescribed fire has long-been touted as a management tool critical for the sustainability of fire-dependent ecosystem services, but with the caveat that it is a dangerous technique that requires considerable planning and careful implementation to ensure safety and control [1]. This notion contains the implicit assumption that fire is more dangerous than other techniques used in natural resource management. No other anthropogenic management technique is as heavily regulated to constrain theoretical ranges of variability in its dynamics and ecological impacts as prescribed fire [2]. Most countries have created laws and policies that impose constraints on when and how fire can be used to meet natural resource management objectives [3], representing a major departure from anthropogenic use of fire in nature prior to the industrialized era [4]. Meanwhile, other management techniques are readily allowed and their relative risk is not questioned with the level of scrutiny used to establish perceptions of risk with prescribed fire. As a result, mechanical techniques are often advocated as tools suitable for replacing fire’s role in shaping ecosystem structure and function [5], despite evidence that mechanical removal provides a negative long-term return on investment for land managers, even when considerable cost-share support is provided [6].

Irrespective of the questionable ecological and economic legitimacy of using mechanical techniques as surrogates to fire, relative measures of risk are needed to provide natural resource agencies with important quantitative information to consider when making decisions that affect landowner use of resource management techniques. To date, decisions have been based largely on perceptions of risk, which can be more reflective of conventional norms or cultural legacies than objective, data-driven information [7]. Understanding perceptions of risk is important; they drive agency support for prescribed burning. Considerably less cost-share support is given for prescribed fire compared to mechanical techniques [8], and isolated fatality events have caused some natural resource agencies to consider reducing their direct involvement in the implementation of prescribed fire on private lands [9]. Studies are therefore needed that test the general perception that fire is a riskier technique relative to other management techniques.

In this paper, we develop first approximations of the relative risk of fatality to private citizens using prescribed fire compared to other management techniques that are readily used in ecosystem management. Due to the complete absence of data capable of answering this question directly (e.g., fatalities or injuries of landowners per unit time of use for various management tools), we use professional occupational fatality statistics as proxies to represent the various techniques used by landowners. Landowners use crop production techniques for weed management, animal production techniques for grazing management, components of logging practices for timber harvest and management of woody invaders, wildland fire techniques to implement prescribed fires in order to enhance forage quality, reduce litter, create structural heterogeneity for wildlife habitat, and prevent/reduce woody invaders, and heavy machinery associated with construction for ranch and farm infrastructural development, general operations, and to reduce woody invaders. We then test for differences in fatalities between prescribed fires and wildfires. Risks of using fire for natural resource objectives in the private sector are almost entirely tied to prescribed fire, not wildfire, and this distinction is not necessarily considered in perceptions of prescribed fire risk. We then identified the factors associated with fatalities in the wildland fire profession and the relative number of fatalities from mechanical (machine-related) and non-mechanical sources.

## Methods

Data were compiled for this study from two databases: the National Interagency Fire Center Wild Fire Accidents by Type of Accident [10] and the United States Department of Labor Bureau of Labor Statistics Census of Fatal Occupational Injuries (CFOI) hours-based fatal injury rates by industry, occupation, and selected demographic characteristics [11]. The WFA database contains a categorized list of fatalities and injuries related to wildfire events. Each entry includes information on the year, place, state, organization, cause of fatality, and number of fatalities for the event. Fatalities are described for each event, whereas a single injury report is filed irrespective of the number of injuries. As a result, we were unable to track the number of injuries resulting from different causal factors and did not include injuries as part of our analysis.

The CFOI database is a collection of six different occupation-specific fatality data sets for 2006 to 2013. The database contains three values: 1) total fatality injuries, 2) total hours worked, and 3) fatality rate. Total hours worked is calculated by multiplying the annual average estimates of total at work US civilians by the average hours for civilians, 16 years of age and older, from the United States Census Bureau Current Population Survey (CPS 2006–2013). The fatality rate represents the number of fatal occupational injuries per 100,000 full-time equivalent workers and was calculated as: (N/EH) x 200,000,000, where N = the number of fatal work injuries, EH = total hours worked by all employees during the calendar year, 200,000,000 = base for 100,000 equivalent full-time workers (working 40 hours per week, 50 weeks per year) (CFOI 2006–2013).

### Proxy measures of fatality risk: occupational fatalities

To develop a first approximation of the relative risk of fatality for different management techniques used by private land managers, we compiled data from each year of the CFOI for occupations that use resource management techniques. Occupational fatality rates served as proxies for relativizing the risks of various land management techniques and included occupations of animal production, operating engineers and other construction equipment operators, crop production, firefighting, and logging workers. Further descriptions of each occupation, including the techniques and practices contributing to hazards in the workplace, are given in Table 1.

**Table 1:** Occupational proxies used to establish the relative fatality risk associated with private citizen use of different management techniques, based on data compiled for different occupations from the Census Database.

Occupational proxy	Comparison to private land management and strength of proxy for comparison
Crop production	Use of heavy machinery and equipment to grow crops for food and fiber; fatality estimates correspond directly to actual fatalities associated with farming.
Animal production	Use of heavy machinery and equipment for animal transportation, ATVs and small equipment for general operations, as well as personal contact with animals; fatality estimates correspond directly to actual fatalities associated with livestock production.
Firefighting	Use of heavy machinery and equipment to suppress wildfires, conduct prescribed fires, and respond to emergencies—including medical, on-the-ground personnel in close proximity to fire for suppression and mop-up procedures; fatality estimates are largely driven by wildfire-related causes, and therefore correspond to higher fatality estimates than would be expected with private land use of prescribed fires.
Logging workers	Use of heavy machinery and equipment to harvest timber for raw material, consumer goods, and industrial products; fatality estimates are associated with commercial operations, whereas landowners are using (1) bulldozers to restore grasslands and savannas following woody plant invasions, and (2) chainsaws and small equipment to fell individual trees. Fatality estimates are therefore expected to be higher for the occupational proxy than on private lands.
Construction equipment operators	Use of heavy machinery and equipment for construction of infrastructure (e.g. roads, structures, ponds); it is unclear how fatality estimates on private lands correspond to professional construction operators given the lack of data for comparisons.

The difference in rates of fatal injuries among occupations was determined using repeated measures ANOVA for years 2006–2013. Students t-tests were used to compute individual pairwise comparisons using the pooled standard deviation. We used Bonferroni adjustment to control the familywise error rate. Data for firefighter fatality rates in 2012 were not included because they were not available.

### Comparing prescribed fire to wildfire fatalities

To compare numbers of fatalities in prescribed fires to wildfires and the causal factors associated with fatalities in each, data were compiled from the WFA database that directly compared wildfire and prescribed fire deaths. These categories, as stated exactly in the WFA Database, included: burnover, burns, entrapment, and snag for both wildfire and prescribed fire (Table 2). Data were only selected from entries between the years of 1963 to 2013 because no entries for prescribed fire fatalities/injuries were found before 1963. This removed a total of 310 fatalities from the wildfire total. Total fatalities from wildfire and prescribed fire were tallied and the mean and standard error of fatalities per year were calculated.

**Table 2:** Comparison of causal factors associated with fire and the post-fire environment accounting for fatality rates in prescribed fires and wildfires from 1963–2014.

Causal factor	Description	Wildfire	Prescribed fire
Burnover	An event in which a fire moves through a location or overtakes personnel or equipment where there is no opportunity to utilize escape routes and safety zones, often resulting in personal injury or equipment damage	140	3
Burns	An injury caused by a cauterizing agent, heat from a fire, or a heated object	9	0
Entrapment	A situation where personnel are unexpectedly caught in a fire behavior-related, life-threatening position where planned escape routes or safety zones are absent, inadequate, or compromised	33	2
Snags	Injury caused by a standing dead tree or part of a dead tree from which at least the leaves and smaller branches have fallen	19	1

### Causal factors of wildland fire fatalities

To determine relative differences in the causal factors accounting for wildland fire-related deaths, irrespective of whether the fatal injury occurred in a prescribed fire or wildfire, data from the WFA database were broadly categorized and separated into mechanical (machine-related) and non-mechanical components (Table 3 shows how we categorized fatal injuries in the WFA database as machine-related or non-mechanical). The data were compiled into four broad categories to establish which components of wildland firefighting contain the highest risk of fatality. The “wildfire” category contained fatalities that were reported as a burnover, entrapment, or fire event. The “vehicle and transportation” category included all events that included the operation of a vehicle including aircraft. The “medical” category contains all events that were injury and/or disease related. The “environment” category contained events that were the result of interactions with the objects in the environment or acts of nature. Two additional categories in the data were not included in the analysis. The “undetermined” category included events with an unknown source of fatality and the “miscellaneous” category contained all events that did not fall into wildfire, vehicular, medical, or environmental where the cause of the fatality/injury was known. Undetermined and miscellaneous categories were not related to sources of wildfire fatalities, or could not be used to track sources of fatalities, so we did not include them in the analysis. We performed locally weighted scatterplot smoothing (LOWESS) to determine the trend in the number of fatal injuries in each category over time.

**Table 3:** Machine-related and non-mechanical causes of wildland fire-related fatalities based on our classification scheme used to summarize the Wildland Fire Accidents Database.

Category	Causal factor	Machine-related	Non-mechanical	Fatality total
Burnover and entrapment	wildfire burnover		X	426
	prescribed fire burnover		X	3
	dozer burnover	X		16
	dozer entrapment	X		0
	engine entrapment	X		2
	wildfire entrapment		X	33
	entrapment prescribed fire		X	2
	equipment burnover		X	0
	patrolling fire		X	0
	tractor plow burnover	X		0
	tractor/tender burnover	X		0
	vehicle burnover	X		1
	vehicle fire	X		1
Vehicles and transportation	aircraft	X		49
	aircraft accident	X		13
	aircraft collision on runway	X		1
	airtanker	X		29
	airtanker accident	X		1
	all-terrain vehicle	X		1
	atv rollover	X		0
	bus rollover	X		0
	contact with aircraft rotor	X		2
	crew carrier rollover	X		0
	crushed by engine	X		1
	dozer rollover	X		4
	driving	X		16
	driving rollover	X		1
	electrocution	X		11
	engine accident	X		0
	engine collision	X		0
	engine hit by train	X		2
	engine rollover	X		22
	heavy engine rollover	X		0
	helicopter	X		56
	hit by vehicle	X		1
	run over by dozer	X		1
	run over by engine	X		6
	run over by vehicle	X		1
	semi-truck	X		1
	smokejumper aircraft	X		1
	struck by motorcycle	X		1
	struck by semi-truck	X		2
	struck by vehicle	X		1
	towing accident	X		1
	vehicle accident	X		58
	vehicle hit by train	X		2
	vehicle left roadway	X		0
	vehicle rollover	X		18
	water tender	X		3
	water tender accident	X		0
	water tender rollover	X		4
Medical	aneurysm		X	2
	asphyxiation		X	3
	wildfire burns		X	21
	heart attack		X	113
	heat exhaustion		X	1
	heat stroke		X	7
	hypothermia		X	1
	medical		X	25
	medical compartment syndrome		X	0
	medical emergency		X	2
	pneumonia		X	1
	pulmonary embolism		X	1
	sickness		X	1
	stroke		X	2
Environmental	drowning		X	4
	fall		X	1
	falling tree		X	1
	fell from engine	X		1
	felling		X	1
	flying debris		X	1
	hazard tree		X	7
	head injury—rock		X	1
	lighting		X	3
	methane gas		X	1
	rock		X	1
	rolling rock		X	1
	smokejumper strangled on let-down		X	1
	tree limb		X	0
	wildfire sang		X	34
	snag prescribed fire		X	1

## Results

### Proxy measures of risk: occupational fatalities

**Fig 1:**
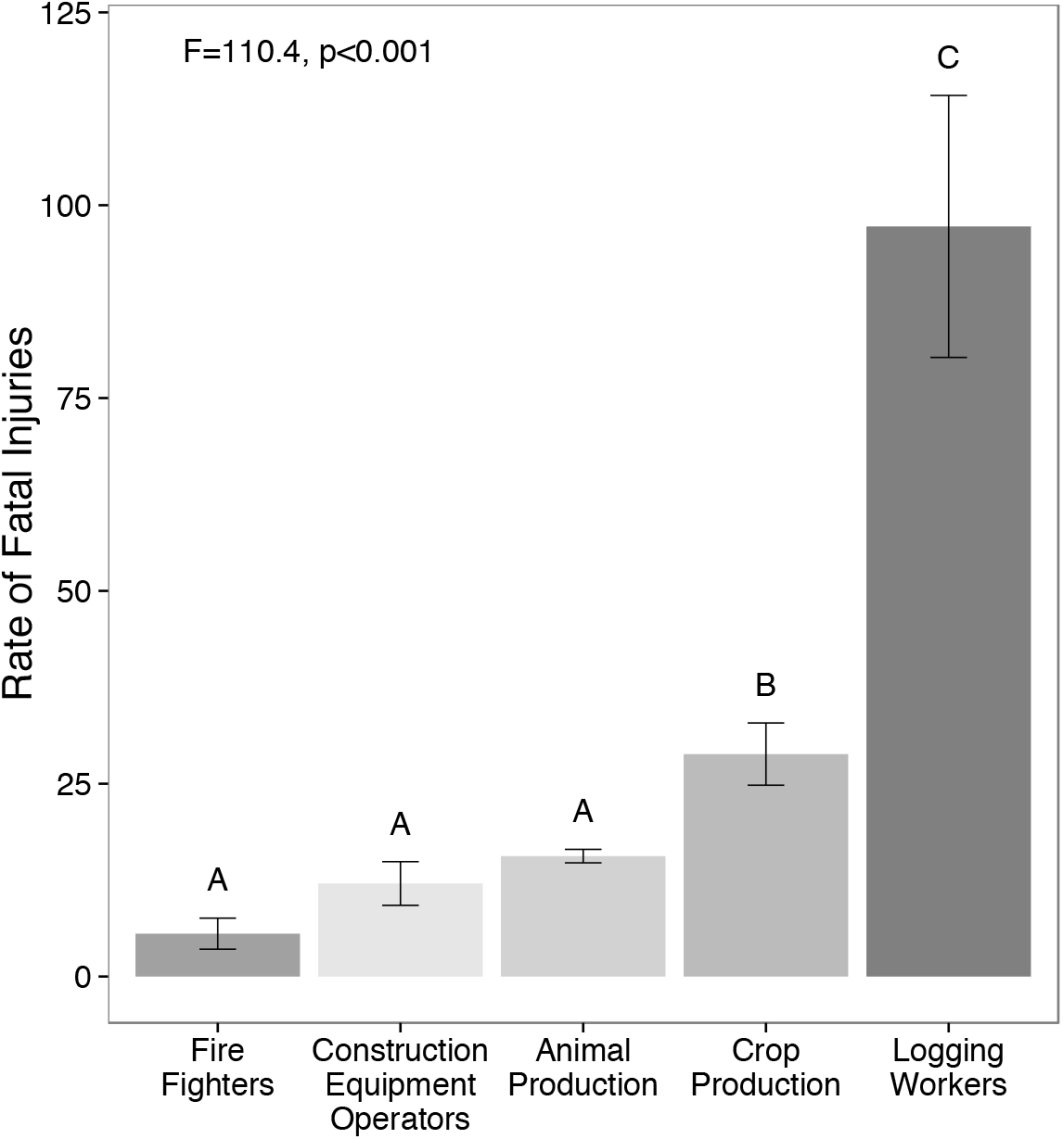
Relative risk of different management techniques used by private land managers, based on occupational fatality rates as proxies. The fatality rate represents the number of fatal occupational injuries per 100,000 full-time equivalent workers. Mean fatality rates and 95% confidence intervals for each occupational proxy for years 2006–2013.

Rates of fatal injuries per 100,000 full time equivalent workers differed among occupations used as a proxy for resource management techniques (F_4,32_ = 110.4, p<0.001). Fire fighting, construction equipment operation, and animal production were not significantly different, with rates of 5.6, 12.1, and 15.6 respectively (Fig 1). Crop production had a higher rate of fatal injury than animal production, fire fighting, and construction equipment operators, with 28.8 fatal injuries per 100,000 full time workers. Logging had the highest rate at 97.3. Both crop production and logging had a higher rate of fatality than fire fighting (Fig 1; students t-test for crop production v. fire fighting: p<0.001; logging v. fire fighting: p<0.001).

### Comparing prescribed fire to wildfire fatalities

**Fig 2:**
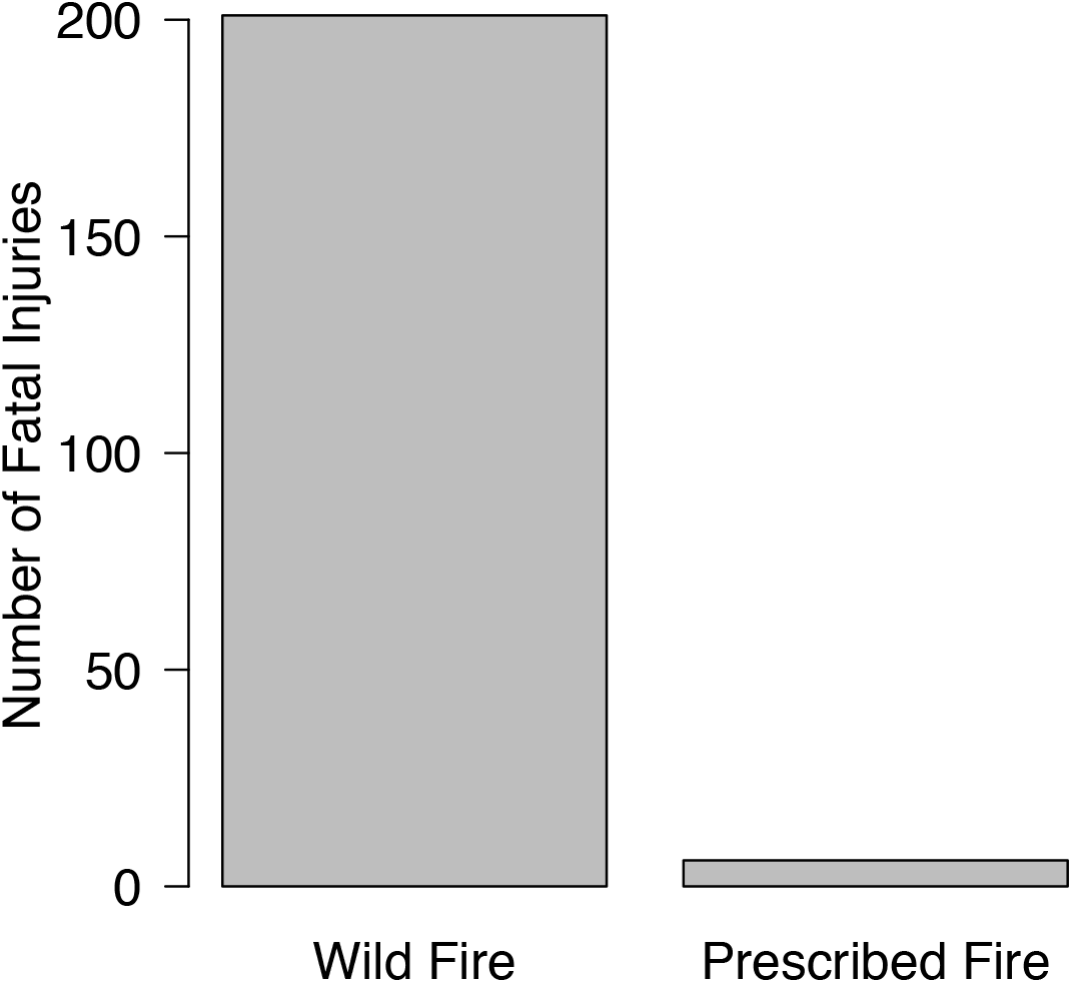
Number of fatal injuries related to wildfire and prescribed fire from 1963–2013.

Fatality risks associated with prescribed fires are substantially lower compared to risks associated with fighting wildfires (Fig 2). Based on data compiled from the WFA database, 201 fatal injuries occurred as a result of wildland firefighting from 1963–2013 (30.4±12.2 per year), compared to 6 from prescribed burning operations (0.12±0.003 per year). Risks of death due to burnover, entrapment and snags are much higher in wildfires than prescribed fires (Table 2), providing evidence that a perception of prescribed fire risk of fatality that is based on wildfire fatality rates is highly erroneous.

### Causal factors of wildland fire deaths

**Fig 3:**
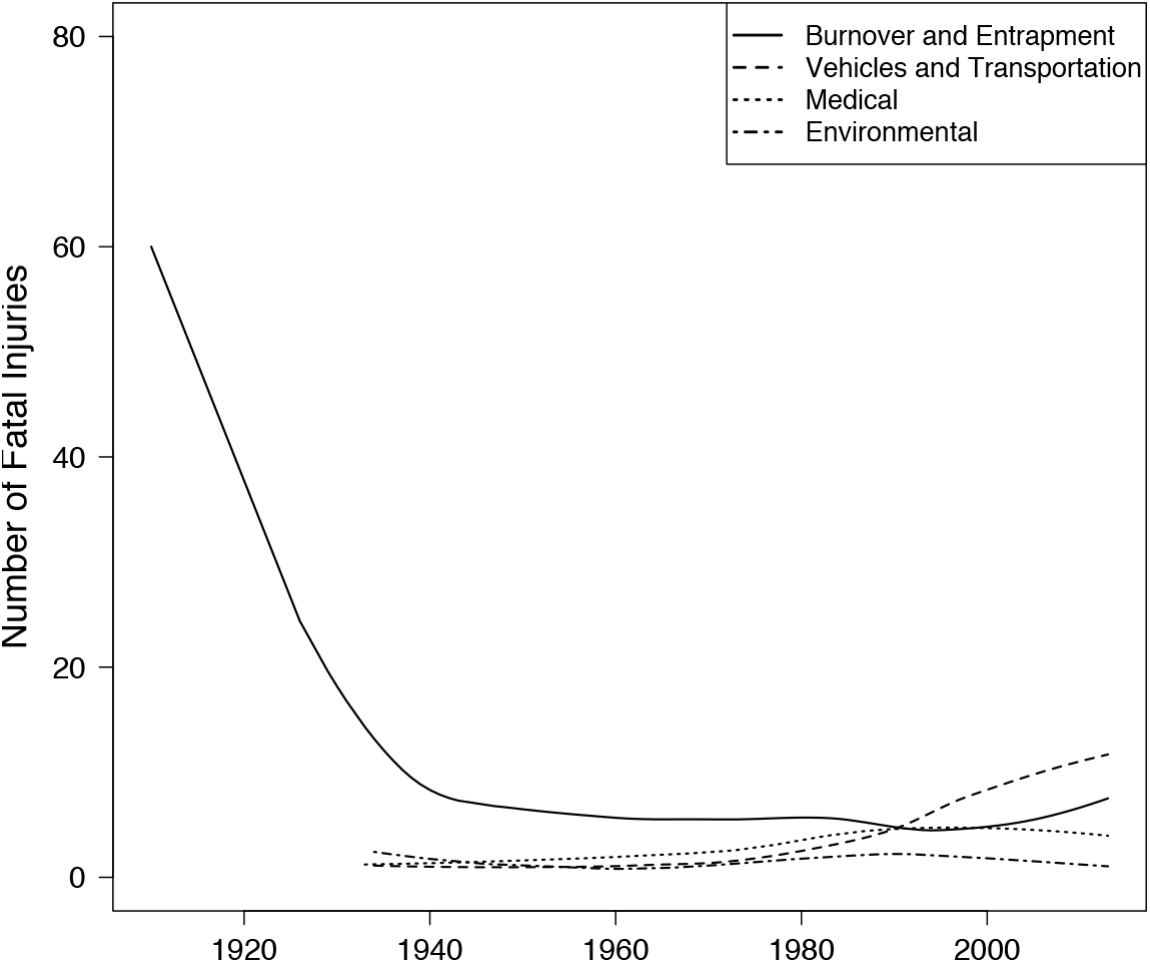
Number of fatal injuries in wildland fire from 1922–2013. (A) Fatal injuries are separated into 4 categories: those directly resulting from fire, those resulting from the use of vehicles and transportation, medical related, and environmental related. Trend in number of fatal injuries over time for each category is represented by locally weighted scatterplot smoothing (LOWESS) of the number of fatal injuries in each category over time.

The factors causing wildland firefighting deaths have changed over the past 90 years. Overall, the number one cause of wildland fire-related deaths since 1913 has been burnover and entrapment. Since 1990, however, vehicles and transportation have been associated with more firefighter fatalities than any other category (Fig 3, more detailed trends over time for all categories of fatal injury shown in appendix A). Before 1990, fatalities related to vehicles and transportation were low, but fatality rates for this category have been steadily rising since the 1980s (Fig 3). In contrast, fatal injuries resulting from burnover and entrapment have dropped precipitously since 1940. Prior to then, the rate of fatal injuries was 60 per 100,000 full time workers, but owed largely to a single data point and high fatality year in 1913. From 1940–2000, the rate of fatal injuries associated with burnover and entrapment has been much lower (less than 10 injuries per 100,000 full time workers; Fig 3A).

## Discussion

Our study provides a step forward in our understanding of the risks to practitioners of prescribed fire in the private sector. Using a combination of approaches including 1) a comparison of fatalities resulting from different occupations that are proxies for techniques employed in land management, 2) a comparison of fatalities resulting from wildland fire versus prescribed fire, and 3) an exploration of causal factors responsible for wildland fire-related fatalities, our data universally suggest that current risk aversion driving the preference for alternative land management techniques over prescribed fire is not supported. We found crop production and logging to have much higher relative risks of fatal injuries than other occupations related to land management on private lands. Firefighting was the least risky of the occupations reviewed herein. Moreover, fatalities from the firefighting profession include wildfire-related injuries as well as prescribed fire-related fatal injuries. In the private sector, risks associated with land management techniques are entirely tied to prescribed fire, not wildfire. This distinction is important when evaluating the risks of using fire in the private sector because wildfire-related fatalities exceed those resulting from prescribed fire by 3,350%.

It is important to recognize that farming and ranching is one of the deadliest occupations in the U.S. workforce. Agriculture, forestry, fishing and hunting-related occupations have consistently been the most dangerous jobs of any industry sector, according to occupational fatality rates from the US Bureau of Labor Statistics. Agriculture involving ranching and farming is one of the ten deadliest jobs in the U.S. [12]. Smith et al. [13] ranked agriculture 2nd of 130 occupations for death rates associated with stress-related diseases (coronary heart and artery disease, hypertension, ulcers and nervous disorders). Consistently, the leading causes of fatalities among ranchers and farmers are transportation and contact with equipment or objects [11]. In contrast, fires and explosions account for less than 2% of fatalities in the agricultural sector every year since the US Bureau of Labor Statistics started keeping track of deaths resulting from fires and explosions, and even fewer of these are likely to be associated with prescribed fires.

The wildland firefighting profession is less dangerous in terms of fatalities than ranching and farming, which is perhaps counter to the perception of natural resource agencies involved with land management on private lands. Relative rates of fatality, based on the number of deaths per number of workers, shows animal production and crop production to be considerably higher than fighting fires. Accidents involving mechanized equipment and vehicles are the leading causes of deaths in agriculture [14]. Tractors and vehicle accidents are the leading causes of fatality for seniors, accounting for over half of all deaths [15]. A similar pattern of risk is evident for youths working in agriculture. Injuries related to vehicles and machinery accounted for over 70% of injuries in youth and were the top reasons for youth fatalities [14]. A similar pattern has emerged in the firefighting profession in recent decades. Over the last 20 years, vehicles, transportation and heavy machinery have been, in general, the leading causes of death. Our findings are supported by other detailed syntheses of firefighter injuries and fatalities. Britton et al. [16] show the most common injury mechanism to firefighters from 2003 to 2007 was slips/trips/falls followed by equipment/tools/machinery.

Calkin et al. [17] describe three common risk biases of agencies involved with fire management: (1) loss aversion: favoring practices perceived to be safe when consequences were framed as potential gains, (2) discounting: favoring reduction of short-term risk over long-term risk, and (3) status quo bias: favoring suppression when suppression was deemed the status quo option. We encourage land management agencies to consider our analysis in the context of these common “biases”. Fatality and injury risks are often predominant concerns for agencies discussing potential support for private lands management programs, so it is interesting to note that natural resource agencies provide great support for programs that require the use of vehicles and heavy machinery and minimally invest in prescribed fire [18]. Loss aversion bias and perceptions of “safe” management actions may explain this discrepancy. Tendencies for discounting long-term risks in favor of short-term risk management are already evident. For example, the decision against using or adequately supporting prescribed fire has contributed to the long-term build-up of volatile fuels and a growing trend toward larger and more severe wildfires. Natural resource agencies have not burned enough land area or conducted a sufficient number of fires to meet their targeted goals, thereby weakening the potential to manage wildland fuels or improve ecosystem health across broad landscapes [19,20]. This pattern of behavior reflects the cautionary tendency and risk averse nature of natural resource agencies, which has greatly limited the application of prescribed fire [7]. Alternatively, our analysis suggests prescribed fire has lower fatality rates compared to other management activities used on private lands. We are therefore left pondering the following question: Is prescribed fire actually one of the safest forms of land management available to resource professionals and private landowners?

Media, imagination and memory, along with social norms and cultural beliefs shape people’s perception of risk and extreme events [21]. For example, surveys suggest urban residents doubt the ability for managers to control prescribed fires and that they will likely escape, posing risks to property and infrastructure [22,23]. This perception conflicts with actual data, which shows 99% of prescribed fires conducted by landowner prescribed burn cooperatives are implemented without incident [24]. This rate of prescribed fire escape is similar to estimates occurring for prescribed fires conducted by federal agencies [22,25]. Thus, very few wildfires result from prescribed burn escapes, and damages and suppression costs are considerably less for prescribed fire escapes, compared to other sources of wildfire ignition and spread [26]. This should be expected; prescribed fire practitioners avoid using fire in weather conditions associated with large and destructive wildfires. The distinction between damages from prescribed fires turned wildfire and other sources of wildfire is important. Agencies are just as concerned with the potential for prescribed fires to escape and cause massive property damage and threaten lives of whole communities than they are with fatality and injury risks to prescribed fire practitioners.

Our analysis is meant to move past the rhetoric and “biases” often associated with decisions regarding prescribed fire use on private lands. Common biases present in fire management have the potential to create fundamental divides between public agencies and private land managers, who tend to prioritize profitability and personal values over risk aversion [27,28]. These differences in values have contributed to increased landowner adoption of prescribed fire in the Great Plains in recent years, largely in response to the loss of productivity and economic profitability resulting from woody plant invasions into grasslands [18]. Differences in problem definition and contrasting consequences to private and public entities have been so great in some regions that private land managers have blatantly disregarded governmentally imposed regulations to implement prescribed fire (e.g. a landowner in TX conducted a burn after failing to get approval for a burn permit [24]). While this example is extreme, we bring it up to showcase that agency decisions to pull support for prescribed fire may lead to counterproductive and surprising decisions among private citizens. Indeed, there is concern that agency support for prescribed burning will constrain landowner burning practices in the central U.S. [18]. For agencies considering lessening technical support for prescribed burning while increasing programs requiring the use of heavy mechanical equipment, our objective data-driven synthesis provides evidence that suggests such an approach may actually increase fatality risks for landowner operators or contractors, not lessen them, and still does not address the long-term risks posed by increasing wildland fuel loadings on private lands.

### Data needs and recommendations

We were able to use existing data sources to develop a comparative first approximation of fatality risk for prescribed fire practitioners in the private sector; however, limitations in data availability constrain the power of assessments attempting to determine the risks of using prescribed fire. Here, we provide a list of recommendations that would improve future evaluations. First, monitoring and data on non-fatal injuries and work-related illnesses are generally inadequate to establish rates of injury and illness associated with land management practices in the private sector [29]. Prescribed fire fatalities, injuries and illnesses are not directly tracked, which precludes direct comparisons with other land management techniques at a national level. One remedy is for private landowners using prescribed fire to establish a safety record of burning activity. This safety record would include, for each fire conducted, the number of injuries, number of fatalities, number of fire-related illnesses, as well as the number of fire escapes (defined as prescribed fires turned wildfire and requiring external help to extinguish the fire; note this differs from a spotfire that is readily extinguished by the crew at hand). Such a safety record is currently available only for some natural resource agencies. Second, documentation of damages from prescribed fire escapes would be useful to test for differences between the relative damage caused by wildfires to prescribed fires that escape. Prescribed fires are typically conducted under very different conditions that help ensure safety and containment of the fire, so one might expect damages to be considerably less for the few prescribed fires that do go awry. Yet, data are so limited that we currently do not know how many prescribed fires are being conducted by private citizens in the U.S., let alone damage estimates, injuries or fatalities associated with prescribed fire escapes. New approaches to collecting these types of data (e.g. involving mobile technology or multimedia data recordings) would greatly improve our ability to provide objective information that compare risks of prescribed fire with other land management tools.

## Supporting Information

S1 Fig Trend in number of fatal injuries over time for A) burnover and entrapment, B) vehicles and transportation, C) medical causes, and D) environmental causes. Lines indicate locally weighted scatterplot smoothing (LOWESS) of the number of fatal injuries in each category over time. Points show number of fatal injuries for each year data are available.
(TIFF)
