# Supplementary figures and images for: First Approximations of Prescribed Fire Risks Relative to Other Management Techniques Used on Private Lands
Source: PLoS One. 2015 Oct 14;10(10):e0140410. doi: 10.1371/journal.pone.0140410 (PMC4605741; doi:10.1371/journal.pone.0140410)

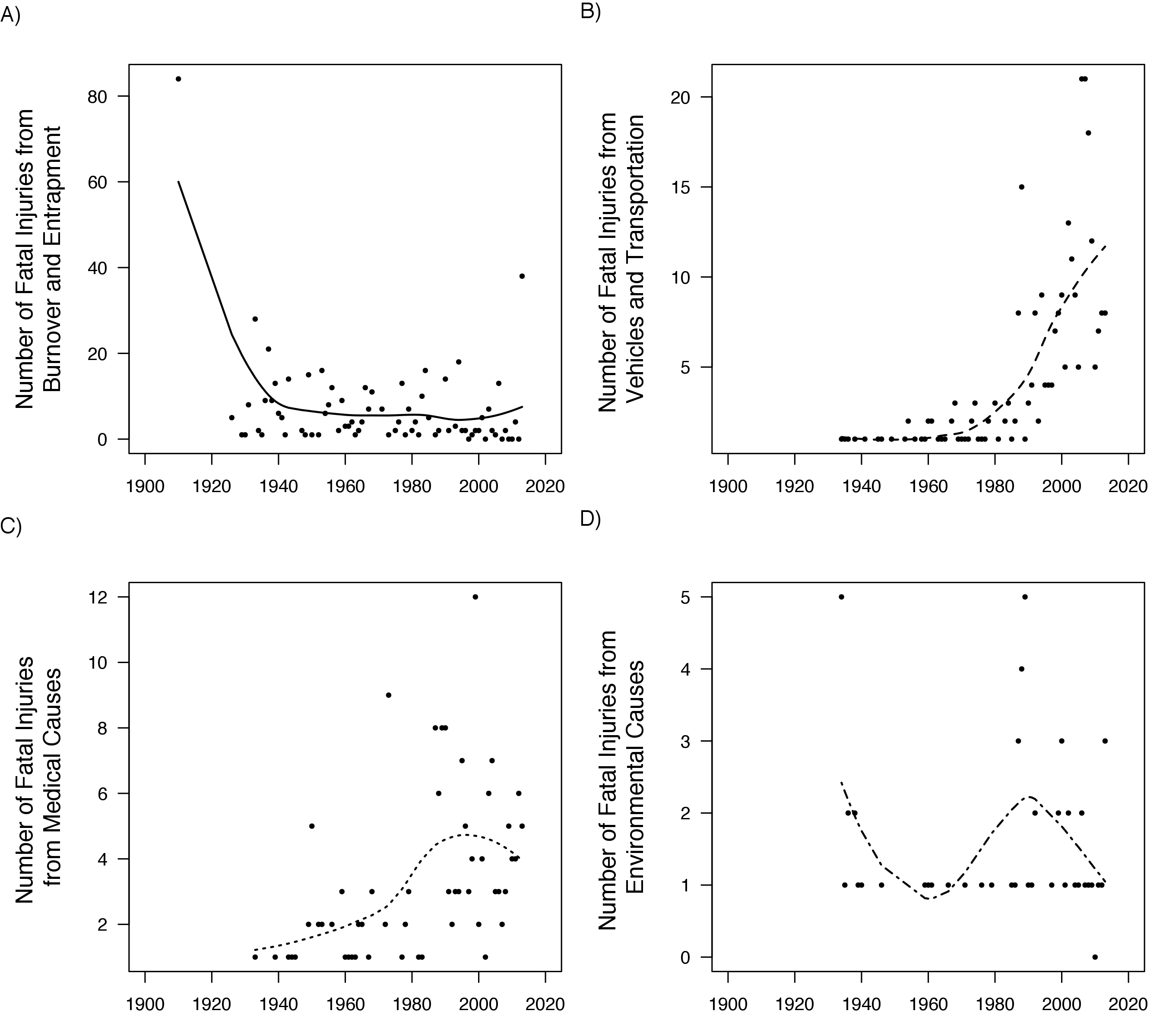

Supplement: S1 Fig — Lines indicate locally weighted scatterplot smoothing (LOWESS) of the number of fatal injuries in each category over time. Points show number of fatal injuries for each year data are available. (TIFF) [file pone.0140410.s001.tiff]
